# PReS-FINAL-2151: HLA B27/B7, TLR-4, NLRP3, CXCR4 AND PTPN12: the shared genetic master-key genes leading to development of undifferentiated spondyloarthritis/era in children

**DOI:** 10.1186/1546-0096-11-S2-P163

**Published:** 2013-12-05

**Authors:** L Lamot, M Vidović, L Tambić Bukovac, F Borovečki, K Gotovac, F Bingula, M Harjaček

**Affiliations:** 1 University of Zagreb School of Medicine, Zagreb, Croatia; 2 Rheumatology, Children's Hospital Srebrnjak, Zagreb, Croatia; 3 Department for the functional genomics, Center for translational and clinical research, University of Zagreb School of Medicine, Zagreb, Croatia

## Introduction

Enthestis related arthritis (era) represent an undifferentiated form of spondyloarthritis (spa), distinguishing it from other subtypes of JIA for optimum treatment and outcome, as well as for studies aimed at understanding genetic predisposition and pathogenesis. Majority of these studies focused on genome associations within MHC, only few searched for connections outside of this region and only some analyzed transcriptome of era patients. Our preliminary results of gene expression profiling in PBMC of 11 new onset and untreated patients diagnosed with era, identified 744 differentially expressed genes.

## Objectives

To validate results of gene expression analysis in groups of patients diagnosed with era and other subtypes of JIA.

## Methods

Based on extensive and diligent statistical analysis, ten genes (CXCR4, NLRP3, PTPRN2, TLR4, DUSP6, MAP2K2, MAPKBP1, MYST3, PTPN12, TNFSF4) were selected for further RT-PCR analysis in four groups of patients selected according to ILAR criteria (N = 56): untreated era patients with obtained expression profile (P1 = 11; 1 B7, 2 B27, 8 B7/B27), untreated era patients (P2 = 10; 2 B7, 6 B27), treated era patients (P3 = 24; 5 B7, 12 B27, 1 B7/B27) and treated JIA patients (P4 = 11). All groups were compared to healthy controls (N = 12). For all era patients HLA typing was preformed.

## Results

Statistically significant (p < 0.05) fold change (FC) was shown for CXCR4 (FC 1.89), TLR4 (FC 4.36), MAPKBP1 (FC -3.13) and PTPN12 (FC -3.70) in P1 group, CXCR4 (FC 1.87), NLRP3 (FC -5), PTPRN2 (FC -5.55), TLR4 (FC 3.91) and PTPN12 (FC -2.63) in P2 group, CXCR4 (FC 1.37), NLRP3 (FC -3.71), MAPKBP1 (FC -1.82), PTPN12 (FC -2.44) and TNFSF4 (FC -1.52) in P3 group and PTPN12 (FC -5) in P4 group.

## Conclusion

Majority of genes with significant FC had a role in NF-κb and MAPK pathways, which are important for regulation of immune response. TLR-4 is known activator of inflammatory cascade that involves NLRP3, and is responsible for secretion of proinflammatory cytokines, while PTPN12 negatively regulates TLR-triggered innate response and lymphocyte activation. Hence, lower expression of PTPN12 can boost immune response. CXCR4 triggers MAPK signaling in response to LPS, and recruits inflammatory cells. CXCR4 showed higher expression in all groups of era patients, regardless of treatment, and PTPN12 showed lower expression even in group of other JIA patients, which could indicate an important role of these genes in pathogenesis of era and other JIA subtypes. TLR4 showed higher expression in untreated era patients, suggesting that standard treatment could influence this gene expression. Interestingly, NLRP3 showed lower expression in group of treated and untreated patients with era. Polymorphisms of NLRP3 that causes decreased gene expression and IL-1β production were linked with increased susceptibility to Chron's disease, while polymorphisms of PTPN12 gene were identified in autoinflammatory diseases. Further on, oncology studies showed PTPN12 gene could be silenced by methylation. Precise mechanism responsible for lower expression of these genes in era and other JIA patients still needs to be clarified.

## Disclosure of interest

None declared.

